# Migrating Oligodendrocyte Progenitor Cells Swell Prior to Soma Dislocation

**DOI:** 10.1038/srep01806

**Published:** 2013-05-09

**Authors:** Patrick Happel, Kerstin Möller, Nina K. Schwering, Irmgard D. Dietzel

**Affiliations:** 1 Central Unit for Ionbeams and Radionuclides (RUBION), Ruhr-University Bochum, D-44780 Bochum, Germany; 2 Department of Molecular Neurobiochemistry, Ruhr-University Bochum, D-44780 Bochum, Germany

## Abstract

The migration of oligodendrocyte progenitor cells (OPCs) to the white matter is an indispensable requirement for an intact brain function. The mechanism of cell migration in general is not yet completely understood. Nevertheless, evidence is accumulating that besides the coordinated rearrangement of the cytoskeleton, a finetuned interplay of ion and water fluxes across the cell membrane is essential for cell migration. One part of a general hypothesis is that a local volume increase towards the direction of movement triggers a mechano-activated calcium influx that regulates various procedures at the rear end of a migrating cell. Here, we investigated cell volume changes of migrating OPCs using scanning ion conductance microscopy. We found that during accelerated migration OPCs undergo an increase in the frontal cell body volume. These findings are supplemented with time lapse calcium imaging data that hint an increase in calcium content the frontal part of the cell soma.

Cell migration plays an essential role in a wide variety of cell types, being crucial for organogenesis, wound healing, immune surveillance and tumor metastases formation. It is regulated by a complex interplay of the actin cytoskeleton dynamics, cell-cell and cell-substrate interactions, transporters, ion channels and aquaporins^1^,^2^,^3^.

Migrating cells are polarised along their axis of movement^4^,^5^,^6^. In most cell types, the leading edge consists of the lamellipodium, a thin, wide and highly motile cell extension^7^. The trailing edge of the cell consists of the cell body containing the nucleus. Migrating cells undergo changes in shape as investigated e.g. by light microscopy in Chinese Hamster Ovary (CHO) and transformed Madin-Darby canine kidney (MDCK-F) cells^8^,^9^.

The conception that local volume changes accompany cell motility evolved from the investigation of the role of ion channels and aquaporins in cell migration^2^,^3^. Ion channels regulate cell volume^10^ and in turn, cell volume regulates the integrity of the cytoskeleton that polymerises within the lamellipodium and thus protrudes the cell towards its direction of movement^11^,^12^,^13^. Furthermore, migrating nasopharyngeal carcinoma cells showed increased volume regulation compared to non-migrating ones^14^.

Among the ion channels, transporters and aquaporins implicated in cell migration the Na^+^/H^+^ ion exchanger NHE1, the Cl^−^/HCO^3−^ anion exchanger AE2 and the aquaporin AQP1 are located at the cell front in fibroblasts, endothelial and CHO cells, respectively^15^,^16^,^17^. The aquaporins AQP4 and AQP9 have been found to enhance lamellipodial activity in astroglial cells and neutrophil granulocytes^9^,^18^ and AQP3 has been shown to be essential for the migration of sperm cells^19^. The influx of Na^+^ and Cl^−^ through transporters has been proposed to cause a local increase in osmotic pressure that is accompanied by water influx through aquaporins and hence local cell swelling^2^,^3^,^9^,^17^,^20^. This increase in local cell volume leads to traction forces in the plasma membrane that might activate mechanosensitive Ca^2+^-channels. This hypothesis is supported by the finding that in migrating keratinocytes and fibroblasts the Ca^2+^-influx is mediated by mechanosensitive channels^21^,^22^,^23^. Calcium channels of the transient receptor potential (TRP) superfamily that activate upon mechanical stimulation have been shown to enhance migration or migration related processes in epithelial cells, spinal neurons and hepatoblastoma cells^24^,^25^,^26^,^27^. Furthermore, it has been shown that local calcium transients mediated by the TRP family member TRPM 7 direct the migration of human lung fibroblasts^28^.

Various reports suggest that the distribution of calcium concentration and its alterations play a role in cell migration. In granule cells, which migrate in a saltatory manner, the rate of calcium transients correlates with the migration velocity and an impairment of the frequency or amplitude of the transients impairs migration^29^,^30^. Furthermore during the migration of neutrophils and fish keratocytes cyclic changes in intracellular calcium concentration have been observed^31^,^32^. In eosinophils, fibroblasts and MDCK-F cells the intracellular calcium is not distributed uniformly but as a gradient with higher Ca^2+^ concentration in the cell body^33^,^34^,^35^,^36^. Neutrophils show a higher calcium concentration at sides of stronger adhesion^37^ and different regions of the cell show different decay kinetics of the calcium transients^38^.

At the trailing edge of the cell, the calcium regulated potassium channel K_Ca3.1_ plays a pivotal role in cell migration. Its blockade slows down migration in epithelial cells, melanoma cells, fibroblasts and microglia^5^,^39^,^40^,^41^,^42^. If internal calcium signaling is switched off the entire cell volume increases as shown by cell volume measurements of fixed MDCK-F cells^24^. The potassium efflux, putatively accompanied by an efflux of chloride, leads to a local cell shrinkage at the trailing edge of the cell as detected by atomic force measurements of living MDCK-F cells^43^. Furthermore, inhibiting volume activated chloride currents also inhibits migration in glioma cells^44^,^45^.

Additionally, theoretical considerations contribute to the concept that volume changes occur during cell migration. Migration is facilitated by an osmotic gradient^46^ and even a simple model omitting intracellular regulation and signaling pathways but including hydrodynamic pressure and swelling stresses exhibits the prominent dynamics of the lamellipodium^47^.

The migration of OPCs has already been investigated in detail by video time lapse microscopy. OPCs move in a saltatory manner alternating between a resting and a moving state with a mean velocity of 10 μm/h ± 7 μm/h and a maximum velocity of 120 μm/h to 140 μm/h (both on poly-L-lysine)^48^. It has been reported that both increases and decreases of the basal internal calcium level impair the migration of OPCs^49^ and that the migration correlates with internal calcium transients^50^. Furthermore, the golli proteins which regulate the migration of OPCs also regulate the expression of the TRP family member TRPC 1^51^ which is proposed to be a component of store operated calcium channels^52^.

To test the hypothesis that an additional mechano-sensitive component plays a role during migration, we first investigated the local changes in cellular volume during random migration of OPCs.

Cellular volume changes were determined using scanning ion conductance microscopy (SICM), a tool to investigate the topography of non-conducting surfaces submerged with electrolyte in a contact free manner^53^,^54^. This method uses the change in access resistance that occurs if an electrolyte filled glass microelectrode is approached towards a non conducting surface to determine the topography of the scanned object. The application of SICM to image living cells has been introduced by imaging melanocytes and human colon cancer cells^55^,^56^. Since then, SICM has been applied to investigate living cells in manifold studies^57^,^58^. Volume determinations by SICM have been validated by confocal light microscopy^59^. Enhanced by a backstep mode it enables one to monitor local volume changes of entire cell bodies^60^,^61^,^62^ as well as to track migrating cells^63^ and is thus a promising tool to investigate local volume changes in moving cells.

Using this method we now detected local swellings in the range of 15% at the frontal part of the soma preceding fast cell body propulsion.

Furthermore, we monitored whether subcellularly segregated Ca^2+^ changes can be detected during the random migration of OPCs. Although calcium sensitive dyes proved to be toxic during long term recordings of these cells or showed a tendency to impede OPC migration, we succeded in detecting subcellular intracellular calcium increases which were localised towards the direction of movement using very small dye concentrations.

Our findings demonstrate for the first time that local water fluxes leading to cell swelling and consequent propulsion of the nucleus occur during saltatory cell migration of OPCs. The observation of local calcium increases further hints at a mechano-sensitive component in the regulation of the internal calcium concentration during OPC migration.

## Results

### Volume changes of mature oligodendrocytes

**Figure 1:**
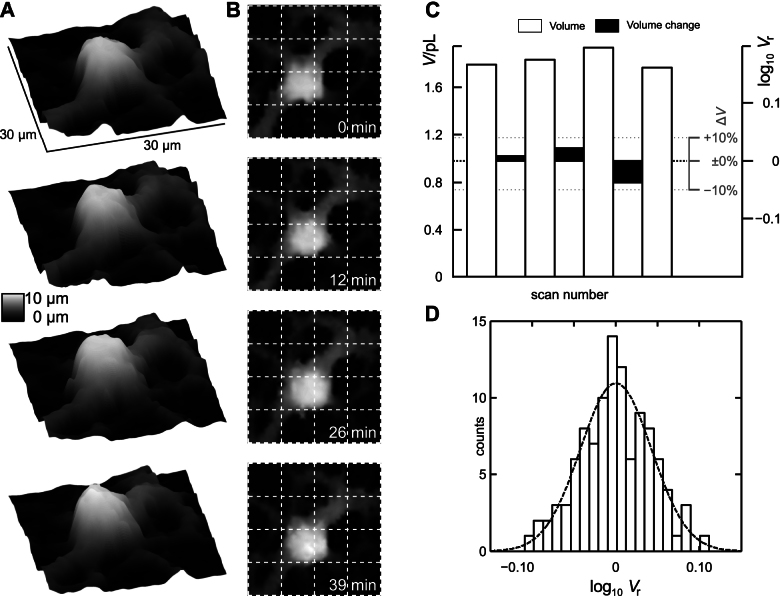
Determination of volume changes in mature oligodendrocytes by scanning ion conductance microscopy. (A,B) Three dimensional (A) and top-view (B) plots of four successive scans of a mature oligodendrocyte. Scans were obtained immediately after the previous scan had finished. Lateral step size was 1 μm, vertical step size was 0.1 μm, scan size was 30 × 30 μm^2^, data shown interpolated by cubic splines. Grey scale indicates height. Note that the oligodendrocyte remained stationary (dislocation amounted to 0.4 μm, 0.5 μm and 0.9 μm, respectively). (C) Calculated absolute (white bars) and relative volume (black bars, logarithmically scaled axis at the right, for easier recognition changes in percent are indicated by the small grey axis) of the cell shown in (A, B). The cell slightly swelled between scans 1, 2 and 3, yielding positive relative volumes and shrank approximately to the volume of scan 1 in scan 4 yielding a corresponding negative relative volume. (D) Histogram of relative volumes of mature oligodendrocytes. Bars represent counts of relative volumes per bin, bin size is 0.011; *n* = 106 calculations of relative volume between two successive scans obtained from 21 different cells. Note that bins represent relative volume at a logarithmic scale. Mean is 0.00 ± 0.04 (SD). Dotted line indicates a gaussian fit to the data, *R*^2^ = 0.86.

Successive scans (127 in total) were obtained from 21 mature oligodendrocytes to investigate volume fluctuations in stationary cells. As an example, three-dimensional plots of four successive scans of an oligodendrocyte are shown in Fig. 1A. The oligodendrocyte remained stationary (migration distances *δ* were 0.4 μm, 0.5 μm and 0.9 μm, respectively, indicated by the plots in Fig. 1B) during the observation time of 39 minutes and only slight changes in cell shape occured. The corresponding volumes *V* and relative volumes *V*_r_ are depicted in Fig. 1C. A slight swelling is visible between scans 1, 2 and 3 (*V*_1_ = 1.79 pL, *V*_2_ = 1.83 pL and *V*_3_ = 1.93 pL) whereas the cell had shrunk back to 1.76 pL in the last scan shown. Accordingly, the decadic logarithm (lg) of the relative volumes (depicted as the black bars in Fig. 1C) is positive between the first three scans and negative between the last two scans (lg *V*_r_ = 0.01; 0.02 and 0.04, respectively, corresponding to +2%, +6% and −9% at linear scale). Fig. 1D shows the histogram of the relative volumes between all successive scans performed (logarithmic scale, *n* = 106, bin size 0.011). Mean value is 0.00 ± 0.04 thus indicating a constant mean volume. The logarithmic representation of the relative volume was chosen because it matches a gaussian distribution (dotted line in Fig. 1D, *R*^2^ = 0.86; in contrast *R*^2^ = 0.79 when the data is plotted at a linear scale). Furthermore, applying a linear scale would assume the same probability for the cell to swell by 100% and to shrink by 100%, thus to disappear completely. In contrast, handling the data using a logarithmic scale confers an equal weight to an increase and a decrease of the cell volume by the same factor which we assume to be more realistic.

The mean migration distance between two successive scans amounted to 0.56 μm ± 1.18 μm. This is below the lateral resolution of the scans^64^ indicating that mature oligodendrocytes maintain stationary.

### Volume changes of oligodendrocyte progenitor cells

**Figure 2:**
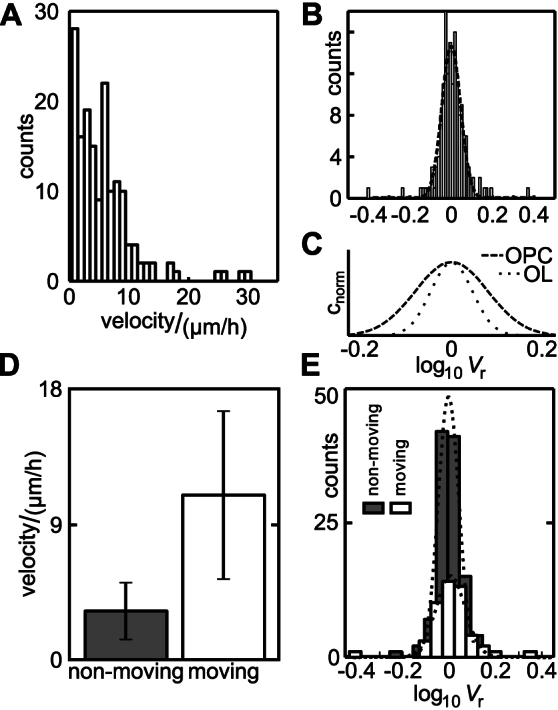
Average velocities and relative volume changes of oligodendrocyte progenitor cells. (A) Histogram of the velocities determined from SICM scans (see Material and Methods section, bin size 1 μm/h; *n* = 161 determinations from 33 different cells; mean velocity: 5.6 μm/h ± 5.1 μm/h (SD), minimum and maximum velocities: 0.17 μm/h and 30.03 μm/h, respectively). (B) Histogram of the relative volume measurements of OPCs. Bars represent counts of relative volume per bin, bin size is 0.011 (as in Fig. 1D), bins represent relative volumes at a logarithmic scale. Mean value: 0.00 ± 0.07 (SD). Dashed line indicates a gaussian fit to the data, *R*^2^ = 0.93. (C) Normalised fits to the relative volumes of adult oligodendrocytes (OL, dotted line, see Fig. 1D) and OPCs (dashed line, see B) on the same *x*-scale. (D) Mean velocities of the cells classified as moving and non-moving. Moving: *v* > 6.5 μm/h, yielding 30% moving cells. Error bars indicate ± SD. (E) Histograms of relative volumes of non-moving (grey bars, *n* = 112) and moving (white bars, *n* = 49) cells. Bin size 0.05, relative volume displayed at logarithmic scale. Mean values: 0.02 ± 0.10 and 0.00 ± 0.05 for moving and non-moving cells, respectively. Dotted lines indicate a gaussian fit to the respective data. *R*^2^ = 0.98 for both fits.

161 pairs of successive scans were obtained from 33 different OPCs. A summary of the analysis of velocity and volume changes is shown in Fig. 2. The histogram of the velocity distribution is shown in Fig. 2A, bin size is 1 μm/h; *n* = 161. Mean velocity amounted to 5.6 μm/h ± 5.1 μm/h, maximum and minimum velocities determined amounted to 30.03 μm/h and and 0.17 μm/h, respectively.

The histogram of the relative volumes (plotted at a logarithmic scale) is depicted in Fig. 2B with a bin size of 0.011 as in Fig. 1D. Mean value was 0.00 ± 0.07. The distributions of the relative volumes of OPCs (dashed line) and adult oligodendroctyes (OLs, dotted line) are plotted in Fig. 2C, clearly showing the larger fluctuations observed in OPCs. However, on the average the volume remained constant. To investigate specific volume changes occuring potentially in migrating OPCs we had to distinguish between moving and non-moving OPCs. A previous investigation of OPC migration had reported a mean velocity of 10 μm/h ± 7 μm/h and a fraction of about 50% of moving cells^48^. Hence, we classified cells as moving if their velocity exceeded 6.5 μm/h. This limit was defined as the mean minus half the SD of the velocity of moving OPCs according to the previous report.

The classification of cells into moving and non-moving cells yielded a fraction of 30% moving cells with a mean velocity of 11.1 μm/h ± 5.7 μm/h. The remaining cells displayed a mean velocity of 3.2 μm/h ± 1.9 μm/h, see Fig. 2D. The mean migration distances between two successive scans amounted to 1.82 μm ± 1.68 μm and 0.43 μm ± 0.66 μm for moving and non-moving cells, respectively. The histograms of lg *V*_r_ of moving (white bars) and non-moving (grey bars) cells are depicted in Fig. 2E. Mean values amount to 0.00 ± 0.05 for non-moving and 0.02 ± 0.10 for moving cells (0% ± 12%; *n* = 112 and 6% ± 27%; *n* = 49, respectively), indicating a slight tendency of the moving OPCs to swell. The larger variance of the moving cells (±0.10) compared with the stationary cells (±0.04 for differentiated oligodendrocytes as well as ±0.05 for progenitor cells) indicates a larger volume fluctuation of migrating cells.

### Local volume changes of accelerating oligodendrocyte progenitor cells

**Figure 3:**
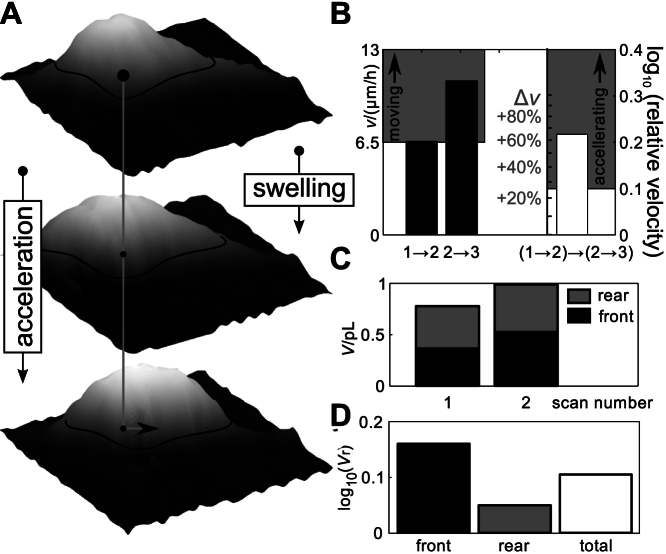
Determination of velocity and volume of an accelerating progenitor cell. (A) Velocity determination of a typical cell included in the migration analysis. Three dimensional plots of a set of three successive scans of a moving oligodendrocyte progenitor cell. Data interpolated by cubic splines, scan size 30 × 30 μm^2^, step size 1 μm laterally and 0.1 μm vertically. (B) Corresponding velocities between scans one, two and three (left diagram). A cell was classified as moving if the velocity between scans two and three exceeded 6.5 μm/h (according to Schmidt et al.^48^). Additionally, a cell was classified as accelerating if the relative velocity exceeded 0.1 at a logarithmic scale (125% in linear representation, right diagram). (C, D) Volume determination. (C) Front and rear cell body volumes of the cell shown in (A). Note that the cell swelling from about 0.8 pL to nearly 1 pL is dominated by an increase in the frontal volume. (D) Corresponding relative volumes of cell body front, rear and total indicating that the cell swelling is dominated by a swelling of the frontal part of the cell.

The hypothesis that cell migration is accompanied by a local volume increase at the moving front of the cell^2^,^3^,^9^,^17^,^20^ was tested by calculating the relative local volumes of cells prior to accelerated migration. Cell volume changes of randomly chosen pairs of migrating OPCs did not significantly differ from cell volume changes of stationary OPCs (see Fig. 2D), as expected if migrating cells oscillate between swelling and shrinkage. To investigate whether systematic volume changes occur during defined time periods of saltatory migration, we now investigated volume changes of cells during the acceleration of migration. To select accelerating cells we analysed sets of three successive scans as shown in Fig. 3A and determined the corresponding velocities. For the example shown in Fig. 3A the corresponding velocities amounted to 6.6 μm/h and 10.8 μm/h, respectively (black bars in Fig. 3B). As defined in Fig. 2 we classified cells with a velocity exceeding 6.5 μm/h as moving (indicated by the grey area in the left diagram of Fig. 3b) yielding 32 scan triples of moving and 96 scan triples of non-moving cells. Additionally, we classified a moving cell as accelerating if the second velocity was at least 1.25 fold of the velocity between the first two scans, corresponding to approximately 0.1 at a logarithmic scale (indicated by the grey area in the right diagram of Fig. 3B; for the example cell lg *v*_r_ amounted to 0.22 corresponding to an increase in velocity by 65%). Nine of the 32 investigated scan triples of moving cells matched the criterion of accelerating cells.

We now investigated whether volume changes occured between the first two scans prior to accelerated migration (see Fig. 3C). To investigate the volume of the front and rear part of the cell body separately the cell was subdivided at the level of *C*_90_, the centroid of the area exceeding 90% of the maximum cell height which approximates the location of the nucleus^65^. The cell shown in Fig. 3A underwent an increase in total cell soma volume from 773 fL to 983 fL (see Fig. 3C) that was dominated by an increase in the frontal soma volume (362 fL to 523 fL, black bars in Fig. 3C). The predominance of the frontal volume increase becomes more apparent in the plot of the relative volumes as depicted in Fig. 3D.

**Figure 4:**
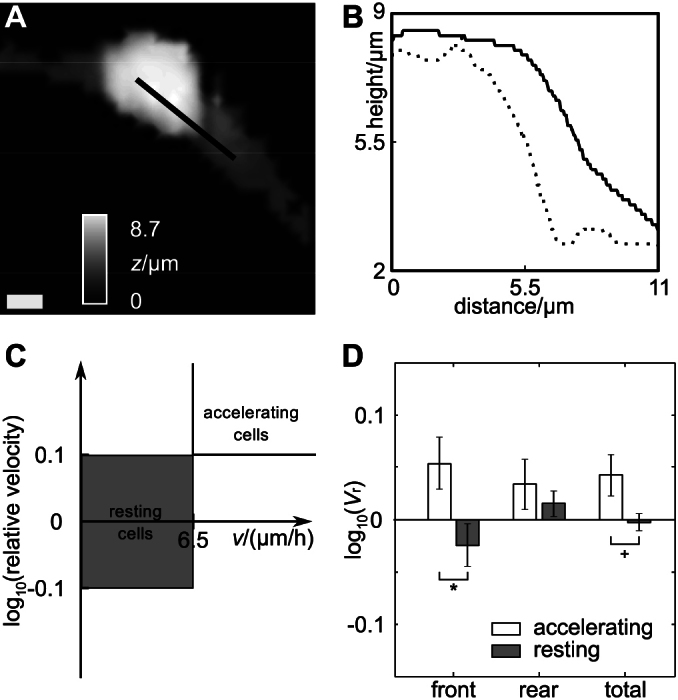
Cell bodies of accelerating cells swell at their frontal part prior to body dislocation. (A) Top view of an SICM scan of an oligodendrocyte progenitor cell. White lateral scale bar represents 3 μm, grey gradient indicates height. Black line indicates the position of height profiles shown in (B). Dotted line represents the profile of the scan obtained at *t* = 0 min, solid line represents the profile of the scan obtained at *t* = 12 min. Note the considerable swelling in the direction of the frontal part of the cell body. (C) Schematic illustration of the definition of accelerating and resting cells. Cells were classified as accelerating when the velocity between scans two and three of the corresponding scan triple exceeded 6.5 μm/h and the relative velocity logarithmically exceeded 1.25 (lg(*v*_r_) > 0.1). Cells were classified as resting if the velocity between scans two and three of the corresponding set of three scans was lower than 6.5 μm/h and the logarithmic representation of its relative velocity ranged between −0.1 and + 0.1. (D) Relative volume of accelerating (white bars, *n* = 9) and resting (grey bars, *n* = 12) cells between the first and second scan of a scan triple (logarithmic representation, error bars indicate SEM). Note that on the average the cell body frontal volumes increased significantly (*p* < 0.05, indicated by the asterisk) and the entire cell body volume with a probability of about 93.5% (*p* < 0.065, indicated by the plus sign) whereas the volume of the rear part of the cell body only showed insignificant changes.

Fig. 4A shows the first scan of a set of three scans from a different cell classified as accelerating. The height profiles of the first two scans (*t* = 0 min and *t* = 12 min) along the black line that corresponds to the direction of movement of the cell are plotted in Fig. 4B (dotted and solid line, respectively). While the height of the profile nearly remained constant, a considerable extension of the cell soma front of more than 2 μm towards the direction of movement is visible.

To investigate whether this local cell soma swelling generally occurs prior to acceleration of migration, we compared average soma volume changes of accelerating and resting cells. Resting cells were defined as all cells displaying a velocity below 6.5 μm/h and a relative velocity in the range of 0.8 to 1.25 (−0.1 ≤ lg *v*_r_ < 0.1 approximately, see Fig. 4C) from our total set of three scans of cells. Twelve out of 96 investigated sets of three scans matched the criteria for resting cells. Scan triples that were neither classified as resting nor accelerating consisted mostly of cells with no detectable increases in velocity and decelerating cells. These cells were excluded from the analysis.

As shown in Fig. 4D accelerating cells (white bars) show a significant frontal cell soma swelling compared with resting cells (grey bars; *p* < 0.05, indicated by the asterisk). On average, the swelling of the frontal part of the soma amounted to 15% ± 7% (0.05 ± 0.03 at a logarithmic scale). Note that here errors indicate ± SEM since we want to illustrate the significance rather than the variation. In contrast, resting cells showed a local front volume decrease of 4% ± 5% (SEM; lg *V*_r_ = 0.03 ± 0.02).

Rear soma volume changes were not significantly different from those of resting cells (accelerating cells: 9% ± 6%; lg *V*_r_ = 0.03 ± 0.02; resting cells: 4% ± 3%; lg *V*_r_ = 0.02 ± 0.01; errors indicate ± SEM) indicating that the observed volume increase prior to cell body acceleration can be mostly attributed to a swelling of the frontal part of the cell body.

### Alterations in intracellular Ca^2+^ fluorescence during migration

To investigate whether the local volume changes might trigger increases in intracellular calcium concentration, we monitored Ca^2+^ transients using the Ca^2+^ sensitive dye Fluo8H-AM with an epifluorescent microscope. From 28 experiments, seven cells clearly identifyable as OPCs showed Ca^2+^ stainings clearly exceeding the background. The large number of failures is due to the low dye concentration required to observe OPC migration, the relatively large magnification used to allow spatial separation of potential Ca^2+^ signals as well as due to drifts of the incubation chamber and the lack of humidification during the recordings. This restricted the data analysed for Ca^2+^-imaging to data recorded during the first two hours of recording. We assume, that at this time the dye had less likely diffused into the cellular compartments, and that the detected signals were thus recorded from the cytosol. From the seven cells analysed, four showed migration.

**Figure 5:**
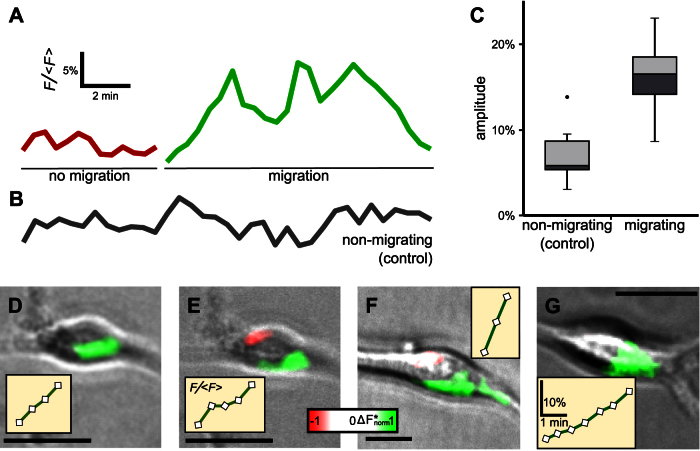
A local Ca^2+^ increase located at the front of the cell body can be observed in migrating OPCs. (A) Fluorescence intensity signal indicating cytosolic free calcium load of an OPC during a phase without migration (red) and during migration (green; note that both traces were recorded from the same cell). (B) Representative trace of a cell showing no migration during the time of observation serving as control. (C) Box plots of the amplitude height of the two largest intensity changes in non-migrating (*n* = 6 from 3 cells) and migrating (*n* = 8 from 4 cells) OPCs. Boxes indicate lower quartile, median and upper quartile, respectively; whiskers indicate maximum and minimum values within 1.5 interquartile ranges (all values beyond this range indicated by dots). (D–G) Superimposition of phase contrast and differences in the fluorescences intensity. The differences are clearly located at the side of the cell pointing towards the direction of movement. Insets show the corresponding amplitude of the cytosolic free calcium load. Scale bars in all images represent 10 μm, all images are flipped such that the direction of movement points towards the lower right of the images. Scale bars in the inset in G apply to all insets. Asterisk in the colour gradient bar indicates the non-linearity due to the applied filter.

Fig. 5A shows changes in the Fluo8H intensity integrated over the entire cell during the random migration of an OPC that showed both a non-migrating (red line) and a migrating phase (green line). As a control, the intensity trace of an OPC showing no migration during the entire recording is shown in Fig. 5B. The Ca^2+^ signal changes during migration clearly exceed the random changes during the non-migrating phase as well as those of the non-migrating OPC. The amplitude of the two largest signal changes of each of the seven OPCs, divided into migrating and non-migrating ones, is shown as box plots in Fig. 5C. Here, the median of the analysed amplitudes is shown as the horizontal line separating the light and dark grey boxes, which represent the lower (dark grey) and upper (light grey) quartile of the data. Thus 50% of the analysed data is located within the box comprising both the light and the dark grey one (interquartile range, IQR). The whiskers represent the minimal or maximal values located within 1.5 IQRs below or above the median, respectively. The median amplitudes of non-migrating (5.8%) and migrating OPCs (16.5%) clearly differ and the upper quartile of non-migrating OPCs (8.7%) does not overlap with the lower quartile of the migrating OPCs (14.1%). While in migrating OPCs all data were within 1.5 IQRs around the median (min: 8.7%, max: 22.8%), we observed an outlier (13.8%, dot in Fig. 5C) in non-migrating cells (min and max within ± 1.5 IRQ: 3.0% and 9.8%, respectively).

Fig. 5D–G show the location of Ca^2+^ signal amplitudes in migrating OPCs. The corresponding amplitudes of the integrated cytosolic free calcium load are shown in the insets (the corresponding scale is shown in Fig. 5G). Although in general Ca^2+^ signals were more homogeneously distributed over the entire cell (not shown), in some recordings in all four migrating OPCs, consistent local Ca^2+^ signal changes were located at the cell side pointing towards the direction of movement.

## Discussion

A local volume increase has been postulated to link ion and water influx at the frontal part of the cell and ion and water efflux at the rear part of the cell by activating stretch-activated Ca^2+^-channels^2^,^3^. Here we succeded to show that in oligodendrocyte progenitor cells indeed a volume change of about 15% can be observed at the frontal part of the cell body prior to accelerated migration.

In contrast to OPCs, on average adult oligodendrocytes did neither alter their position nor volume. The observed deviation of ± 0.04 (Fig. 2) in cellular volume most likely includes both physiological volume fluctuations of the oligodendrocytes and uncertainties of the volume determination by the instrument. Furthermore, as mentioned in the Materials and Methods section, theoretical considerations estimated the error in the determination of the ratio of two volumes to amount to 8%. Thus, we conclude that volume changes exceeding 10% (that is 0.04 in logarithmic scale) on the average are not a result of the uncertainties of the applied determination method of the volume changes, but represent true cell swellings.

OPCs on average did not alter their volume systematically, but showed larger fluctuations than mature oligodendrocytes. Since it is known that only a fraction of OPCs migrate^48^,^49^,^50^ we devided our set of recordings into migrating and non-migrating cells by applying a threshold of 6.5 μm/h. This is in the range of the threshold for migrating cells defined previously^50^ and furthermore represents the mean minus half of the SD of the velocity reported by Schmidt et al^48^. This threshold ensures, that no stationary cell was counted as migrating. Since the determination of the position of the OPC is based on the centroid of multiple pixels, the systematic error does not exceed the size of a pixel which thus served as an upper boundary. A pixel size of 1 μm and a corresponding acquisition time of 10 min results in a maximum error of 6 μm/h. This threshold yielded a slightly smaller fraction of migrating cells than previously reported under similar conditions^48^. The smaller fraction of migrating OPCs is due to limitations of the SICM setup presently used. Since the cells were positioned in the center of the scanning frame, a distance of approximately 15 μm could be migrated by a cell without leaving the area of observation (resulting from half the diagonal of the scan area minus half of the cell body length (approx. 10 μm)). Additionally, we only investigated the cells that were located within the scan area for a period of at least three scans to determine changes in velocity. Hence, cells faster than 30 μm/h were discarded and do not contribute to the fraction of migrating cells analysed here. This approximation matches with the maximum velocity determined by SICM of 30.03 μm/h. During our investigations, we observed additional 19 cells that were discarded due to this limit.

While on average the volume of non-migrating OPCs was constant with a standard deviation similar to that of adult oligodendrocytes, migrating OPCs showed a slight volume increase and a larger standard deviation. This indicates that in migrating OPCs larger volume fluctuations occur.

OPCs migrate in a saltatory manner^48^,^50^, thus accelerating and decelerating. In phases of migration with small changes in velocity, potential volume increases and compensating decreases might not be detectable with the temporal resolution of the SICM presently used. Thus, only recordings of accelerating OPCs were evaluated. OPCs classified as accelerating showed a significant volume increase towards the direction of movement prior to accelerated migration if compared to resting cells. To our knowledge, our results are the first observations of a local volume increase towards the direction of movement confirming the occurence of the water fluxes postulated in a general model of cell migration^2^,^3^,^17^,^18^,^20^.

If, as postulated by the model, the local volume increases trigger a mechano-sensitve influx of Ca^2+^, a local increase in intracellular Ca^2+^ should be observable as well. Indeed, increases in the fluorescence intensity of the calcium binding dye Fluo-8H that exceeded the Ca^2+^ signal changes of non-migrating cells could be observed in randomly migrating OPCs. This is in good agreement with previously published data showing that migration velocity and intracellular Ca^2+^ activity correlate in OPCs^50^. In addition, we showed that during the random migration of OPCs, cytosolic free Ca^2+^ load increases in the range of 10% to 20% could be observed that were mainly located at the side of the cell body pointing towards the direction of movement.

Since OPCs died within the first hour after staining with the ratiometric dye Fura-2 and showed no migration if stained with a mixture of Fluo8-H and Calcein blue, ratiometric determinations of the calcium signal could not be recorded. In contrast to ratiometric measurements, the observed signal, which was integrated over the entire cell, does not represent the intracellular calcium activity, but the cytosolic free calcium load. Nevertheless, the spatial distribution of the calcium signal most likely comprises both volume changes and thus spatial increases in sample thickness and increases in the cytosolic calcium activity. Since the subcellular increases in cytosolic free calcium load which were located at the side of the cell pointing towards the direction of movement were accompanied by an increase in the cytosolic free calcium load integrated over the entire cell, it can be excluded that they were caused solely by volume changes. A local increase of the Fluo8H signal due to a local volume increase without accompanying calcium fluxes would instead be accompanied by a constant or decreasing signal of the calcium load of the entire cell.

Assuming a volume increase of 11% of the entire cell as determined by the SICM measurements, the observed increases in cytosolic free calcium load of 10% to 20% indicate that the increased volume does not lead to a decreased calcium activity, but is compensated either by Ca^2+^ influx or release from internal stores. Furthermore, assuming that the total calcium content increase occurs only at the frontal part of the cell (20% to 40% increase at the frontal part of the cell), which swells by 15% as detected by SICM measurements, the resulting calcium activity increase would be in the range of 4% to 22% (further assuming equal frontal and rear volumes before swelling and a linear relationship between fluorescence intensity and calcium activity). Although this is a very rough estimation, it hints at an increased calcium activity at the frontal part of migrating OPCs that might be mediated via mechano-sensitive channels.

However, since we found that ratiometric measurements with OPCs were not realisable, detailed analysis of the spatial calcium changes during OPC migration would require the development of a more sophisticated instrument combining SICM and confocal methods as well as a migration assay with a lower failure rate.

Furthermore, since OPCs stained with Fluo8H-AM did show practically no migration at room temperature, we observed Ca^2+^ fluctuations during random migration at 37°C. Under these conditions and with an increased external calcium concentration as well as supplements of PDGF and bFGF, we observed migration velocities of up to 150 μm/h, which is in good agreement with previous reports^48^,^50^. Nevertheless, as explained above, only migration velocities up to 30 μm/h could be recorded with our presently available SICM, rendering simultaneous determination of both internal Ca^2+^ and cellular volume changes impossible. Cells which migrated faster than 30 μm/h were not included in our SICM analysis.

Although we could not directly link temporal sequences of local volume and local calcium activity increases with the instrumentation presently available, our data show that both phenomena occur during random migration of OPCs. Since OPC migration has been shown to be correlated with intracellular Ca^2+^ alterations mediated by voltage operated calcium channels (VOCCs)^50^, our data can be interpreted in two ways: Firstly, the voltage activated increase in intracellular calcium could induce the observed volume increase, secondly, the local increase in intracellular Ca^2+^ triggered by the volume increase through mechano-sensitive ion channels might create the initial depolarisation required to trigger a subsequent Ca^2+^ influx via VOCCs.

If one considers the ratio of internal Ca^2+^ and particularly K^+^ concentration, which is in the range of 10^−3^ to 10^−4^, an increase of Ca^2+^ by several per cent seems unlikely to cause an osmotic effect per se. However, Ca^2+^ might induce reactions that increase intracellular osmolarity, either by changing the electric potential which in turn induces ion influxes or via its role as a mediator in manifold biochemical signalling cascades.

The hypothesis that the volume increase triggers a calcium increase via mechano-sensitive ion channels is supported by several observations. Firstly, if VOCCs are blocked using verapamil or nifedipine, a basal movement of OPCs remains, although severely reduced, but blocking external Ca^2+^ by the addition of EGTA instead exceeds the effect of VOCC blockers^50^.

Secondly, OPCs are known to express several ion channels and transporters that are supposed to play a role in the regulation of the osmotic pressure during migration (see introduction for details), although one should consider that not all OPCs express the same set of ion channels^66^. OPCs are known to express both a Na^+^/H^+^ and a Cl^−^/HCO^−^ exchanger^67^, suggested to be the mediator of a local increase in osmotic pressure^2^,^3^,^17^,^18^,^20^. Futhermore, a Ca^2+^ dependent potassium outward current is likely to mediate a compensatory volume decrease at the rear end of the cell^5^,^39^,^40^,^41^,^42^,^68^. Potassium currents have been investigated in OPCs. Both, spindle shaped OPCs as investigated in this study and OPCs in a later phase of development show similar potassium outward currents. In later OPCs, some of these currents have been shown to depend on internal Ca^2+^ 69.

Although a sole efflux of K^+^ might be sufficient to cause a compensatory volume decrease at the rear end of the cell, most likely an accompanying efflux of Cl^−^ occurs, since the hyperpolarisation by the K^+^ efflux would support chloride efflux. Virtually nothing is known about chloride currents not activated by ligands in OPCs. Nevertheless, a large intracellular Cl^−^ concentration is found in OPC descendants^70^. Furthermore, both astrocytes and glial cell derived tumor cells express volume regulated chloride channels^45^,^71^,^72^.

In our report, we showed that scanning ion conductance microscopy is an excellent tool to quantify cellular volume changes during cell migration in culture. The application of this emerging technique to observe randomly migrating OPCs revealed for the first time that local volume changes at the frontal part of the cell body, hence in the direction of movement, occur during accelerated migration of OPCs. Recent improvements of this technique^73^ allow the observation of flat samples such as the lamellipodium with high spatial and temporal resolution, potentially enabling the observation of volume changes in cellular structures where volume relevant proteins are located.

Furthermore, we observed local increases in the internal calcium activity that, too, were located at the frontal part of the cell body of migrating OPCs. Albeit various interpretations of these observations are conceivable, our findings agree well with the postulated model that a local volume increase triggers a succeeding increase in internal calcium concentration. Notwithstanding that an exact determination of the causal link between volume changes and Ca^2+^ surges is still lacking, our data contribute a yet missing aspect to unravel the mechanism of cell migration.

## Methods

### Cell culture

Cells were obtained and cultured similarly as described previously^74^. Briefly, after passing the brains of two to four days old Sprague-Dawley (SICM observations) or Wistar (Ca^2+^ imaging) rats through nylon meshes of 125 μm and 36 μm pore size, the cells were collected in phosphate buffered saline (PBS, containing in mM: 138 NaCl, 8.1 Na_2_HPO_4_, 2.7 KCl, 1.47 KH_2_PO_4_) and centrifuged for 10 minutes at 900 rpm and 4°C. The cells were then resuspended in glial mixed culture medium (GMM, consisting of a mixture of HAM's F12 and DMEM (1:1) supplemented with 10% fetal calf serum, 100 U/mL penicillin and 100 μg/mL streptomycin) and pre-cultured in cell culture flasks (in humidified air containing 5% CO_2_ at 37°C) until a dense layer of cells had grown. Cell debris and the different glial cell types then were separated by their different adherences to the flask: To remove microglia and debris flasks were shaken for 3 hours at 180 rpm. The supernatant was replaced by fresh GMM and the cell culture flasks were shaken for additional 16 to 18 hours. The supernatant now containing OPCs was centrifuged for 5 min at 1700 rpm at 4°C. The pellet was resuspended in GMM and preplated for 45 minutes in uncoated cell culture dishes to remove cells that were not OPCs but had been detached by the shaking procedure. The supernatant containing the non adherent cells was diluted to approximately 50000 cells/mL. In dependence on the further experiments, cells were then treated differently as detailed below.

### Scanning ion conductance microscopy

For SICM measurements, 500 μl of the the cell suspension were transferred into removable glas rings (diameter 1.5 cm) which had been placed in the center of poly-L-lysine coated plastic cell culture dishes (diameter 3.5 cm). After one to three hours the rings were removed and medium was exchanged to 1 mL GMM.

SICM measurements were performed using the same instrument as previously described operating in floating backstep mode^60^. Scans were performed in 3.5 cm plastic petri dishes after exchanging the medium to Leibovitz-15. In order to reduce migration speed with the aim of preventing cells from escaping out of the scanning frame, neither basic fibroblast growth factor (bFGF) nor plateled derived growth factor (PDGF) was added to the culture medium.

Scanning electrodes were filled with extracellular saline (containing in mM: NaCl 110, KCl 5.4, CaCl_2_: 1.8, MgCl_2_ 0.8, Glucose 10, HEPES 10). Inner diameter of the scanning probes was about 1 μm, access resistance was about 4 MOhm, scan size was 30 μm × 30 μm with a lateral step size of 1 μm and a vertical step size of 100 nm. These step sizes allow recordings with a voxel volume in the same range as the volume of the confocal spot of a confocal microscope. Acquisition time was about 10 minutes per scan. Data are shown interpolated by cubic splines.

### Volume determination

Volume calculations of mature oligodendrocytes were performed as described previously^60^ by calculating the sum of the volumes of each single column above each pixel: *V* = Δ*x*Δ*y*Σ*_i_z_i_*; where Δ*x* and Δ*y* denote the lateral scan step size and *z_i_* denotes the detected height of the pixel *i*. Volume calculations of OPCs were performed by applying a boundary delimitation algorithm (BDA) as detailed in^65^. This method circumvents the problems that occur in the separation of processes from cell somata by defining a certain height threshold^74^,^75^. In brief, after aligning the cell parallel to the *x*-axis polynomials of third degree were fitted along the contour of sections parallel to the *x*, *z*-plane of the cell. The cell was subdivided at the level of *C*_90_, a single data point used to approximate the location of the nucleus. It was defined by the centroid of the area covered by the pixels exceeding 90% of the maximal cell height.

Relative volumes were calculated as *V*_r,*n*_ = *V_n_*_+1_/*V_n_* where *n* denotes the scan number. The influence of the lateral step size on the volume determinations was investigated by simulating scans of increasing step sizes on the basis of scans with small step sizes. The use of a step size of 1 μm led to an underestimation of the volume by 8% ± 4%; *n* = 9. While the underestimation is cancelled when calculating the ratio of two volumes, the error of 4% duplicates. Therefore we estimate that the determination of the ratio of two volumes is subject to a systematic error of 8%.

### Velocity determination

Cell body position was approximated by *C*_20_, the centroid of the area exceeding 20% of the maximal cell height. Its coordinates were calculated as described before^65^. Velocities were calculated as *v_n_* = *d_n_*/Δ*t_n_* where *d_n_* is the distance between the positions of *C*_20_ of the corresponding scans *n* and *n* + 1, Δ*t_n_* denotes the time interval between the corresponding scans. For the calculations of the average migration distance, the direction of movement was taken into account. We defined the longest distance that was migrated without a change in direction as positive.

### Data processing

SICM data were processed using Matlab (R2008a) and ImageJ (1.42q) software. Statistical calculations were performed in R (2.7.1). Tests for significance are Welch two sample *t*-tests. Errors indicate standard deviations (*σ*) unless otherwise noted. Standard error of the mean was calculated as 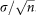.

Before applying the BDA the SICM data was filtered using a threshold filter clipping every *z*-value below 1 μm to zero and plane corrected when necessary.

### Ca^2+^ imaging

One milliliter of the cell suspension (see section Cell Culture) was transferred into poly-L-lysine coated plastic cell culture dishes. After adherence of the cells, medium was changed to proliferation medium (PM, containing DMEM, 1 × B27 (Gibco), 10 ng/ml PDGF, 10 ng/mL bFGF, 2 mM glutamin, 100 U/mL penicillin).

We tested ratiometric methods to investigate the calcium activity. However, OPCs stained with both Fluo-8H and Calcein blue did not show migration and OPCs stained with Fura-2 quickly died within one hour after staining. Thus, for Ca^2+^ recordings, cells were stained with 0.5 μM Fluo8H-AM in PM for 30 minutes at 37°C, 5% CO_2_ in humidified air. After rinsing with PBS, the medium was exchanged to fresh PM. Here, in contrast to SICM investigations, a medium containing growth factors was used since otherwise the Fluo-8H stained cells did not migrate. The intracellular Ca^2+^ activity during the random migration of OPCs was observed with an Olympus IX81 inverted microscope (FITC channel) using a 20fold objective, equipped with an incubation chamber (H.Sauer Laborbedarf, Germany), at 37°C and 5% CO_2_. Fluo8H intensity was recorded with a monochrome CCD camera equipped with an ICX 0285 sensor (Sony) featuring 1376 × 1032 pixels. No additional binning was applied. Exposure time was 1000 ms, images were recorded during four to six hours at 30 s intervals.

Fluo-8H intensity was analysed within a ROI comprising the entire cell during the time of observation, thus integrating over the entire cell volume and therefore limiting the volume dependence of the fluorescence signal to the activity depending binding properties of the dye. The fluorescence signal in this case represents the free calcium load of the cell.

The use of a single calcium sensitive dye as well as an epifluorescent microscope limits the interpretation of the Ca^2+^ recordings. Under these recording conditions, changes in the fluorescence signal can occur due to several causes: First, a change in intracellular Ca^2+^ activity is induced by transmembrane Ca^2+^ fluxes or release/uptake from intracellular stores at constant cell volume. Second, a volume change of the cells would affect the fluorescence signal in a ROI comprising the entire cell as follows: A sole redistribution of volume would lead to no changes in the signal since a volume and thus sample thickness increase at one position would be compensated by a local volume and thus sample thickness decrease at a another position. A volume increase without changes of the cytoplasmatic free Ca^2+^ load would lead to a dilution of both, Ca^2+^ and dye. Even if the activity dependence of the fluorescence intensity is neglected, this could at maximum lead to a constant signal integrated over the entire cell, but not an increase in intensity. Therefore, an increase in the fluorescence signal within a ROI comprising the entire cell can only be interpreted as a net cytosolic increase in free Ca^2+^ load. However, these recordings do not allow to determine the contribution of volume and calcium activity changes to the change in the fluorescence intensity signal.

Calcium recordings were corrected for background and bleaching, assuming an exponential decay, calcium signals were normalised to the mean intensity of the entire recording (*F*/〈*F*〉). Local alterations in the intracellular calcium level were calculated by subtracting the corrected fluorescence signal of two respective recordings. For easier visualisation of the localisation the differences were filtered by applying a smooth bandpass filter in the frequency domain, cutting off signals the size of which was below 3 px to 15 px and above 40 px to 100 px and subsequently normalised to the maximal difference (Δ*F*_norm_, see Fig. 5). Note that after the application of that filter colour gradients do not represent the difference in calcium linearly. Amplitudes (Δ*F*/〈*F*〉) were determined as the maximum increase between two recordings within a time period of three minutes.

## Author Contributions

PH and IDD designed the study. KM, PH and NKS recorded and analysed the data. PH and IDD wrote the manuscript. All authors reviewed and approved the final manuscript.
